# Single DNA Translocation and Electrical Characterization Based on Atomic Force Microscopy and Nanoelectrodes

**DOI:** 10.1109/ojnano.2022.3217108

**Published:** 2022-10-25

**Authors:** BO MA, JIN-WOO KIM, STEVE TUNG

**Affiliations:** 1 Department of Mechanical Engineering, University of Arkansas, Fayetteville, AR 72701 USA; 2 Department of Biological & Agricultural Engineering, University of Arkansas, Fayetteville, AR 72701 USA; 3 Materials Science and Engineering Program, University of Arkansas, Fayetteville, AR 72701 USA; 4 Institute for Nanoscience and Engineering, University of Arkansas, Fayetteville, AR 72701 USA

**Keywords:** Atomic force microscopy, DNA linearization, DNA translocation, single DNA control

## Abstract

Precision DNA translocation control is critical for achieving high accuracy in single molecule-based DNA sequencing. In this report, we describe an atomic force microscopy (AFM) based method to linearize a double-stranded DNA strand during the translocation process and characterize the electrical properties of the moving DNA using a platinum (Pt) nanoelectrode gap. In this method, *λ*DNAs were first deposited on a charged mica substrate surface and topographically scanned. A single DNA suitable for translocation was then identified and electrostatically attached to an AFM probe by pressing the probe tip down onto one end of the DNA strand without chemical functionalizations. Next, the DNA strand was lifted off the mica surface by the probe tip. The pulling force required to completely lift off the DNA agreed well with the theoretical DNA adhesion force to a charged mica surface. After liftoff, the captured DNA was translocated at varied speeds across the substrate and ultimately across the Pt nanoelectrode gap for electrical characterizations. Finally, finite element analysis of the effect of the translocating DNA on the conductivity of the nanoelectrode gap was conducted, validating the range of the gap current measured experimentally during the DNA translocation process.

## I. INTRODUCTION

Single molecule-based DNA sequencing techniques, such as nanopores and nanochannels, are among the most promising approaches for portable genome analysis [1], [2], [3]. Unlike the ‘sequencing-by-synthesis’ method employed by most commercial sequencing services, nanopores and nanochannels utilize nanoscale sensing elements to characterize the electronic signature of individual nucleotides [4], [5], [6]. In nanopore sequencing, DNA strands are pulled through a nanopore by an electrophoretic force and the variation in the ionic current through the pore is used to establish the DNA sequence. The protein nanopores manufactured by Oxford Nanopore Technologies (Oxford, United Kingdom) utilize enzymatic ratcheting to control the translocation of DNA through a nanopore. In this process, an enzyme binds to a sample double-stranded DNA, unzips it into two single-stranded DNAs, and then ratchets one of the ssDNAs through the nanopore. The DNA ratcheting process, unfortunately, is stochastic due to the nature of enzyme turnover, which leads to different time scales for the ratcheting bases [7]. When the time scale is too short, the ionic current signal becomes burried in the background noise. When it is too long, the signal might not be distinguishable from a repetitive sequence of identical bases. To date, enzymatic ratcheting, together with device spatial resolution, remain the key technological challenges in nanopore and nanochannel based sequencing [8]. The spatial resolution of the nanopore is dictated by the diameter and thickness of the pore. By using a two-dmensional material, such as graphene and MoS_2_, to fabricate the pore, it is possible to minimize the pore thickness and thus maximize the spatial resolution [9]. However, previous studies indicated that the ionic current of the nanopore is affected not only by the bases inside the pore but also those in the immediate viscinity of the pore structure [10]. Therefore, while the use of two-dimensional material is advantageous, it might not be the ultimate solution for achieving high spatial resolution. Instead, the incorporation of a separate nanoscale sensing element that measures the electrical property ‘across’ the backbone of the translocating DNA might provide significant benefit when compared to the ionic current method.

Regulating DNA translocation has been investigated from multiple perspectives including modifying the DNA structure and manipulating the experimental conditions such as salt concentration, driving voltage, and dimensions of the translocation path [11], [12], [13], [14]. These methods are mostly designed to regulate DNA translocation indirectly and therefore highly susceptible to environmental conditions. Direct manipulation techniques, on the other hand, rely on direct physical contact with the DNA. For example, prior studies have successfully tethered DNA to a nanopositioning system to lower the translocation speed in order to improve the temporal resolution of a nanopore [15], [16]. While feasible, this approach relies on probe tip functionalization to achieve DNA tethering and it is difficult to limit the tethering to a single DNA strand.

AFM is an effective tool for DNA manipulation due to the close matching between the physical dimensions of DNA and the spatial resolution of AFM. Using a task-specific probe tip, DNA can be stretched, indented, and cut-and-pasted by a piconewton level force [17], [18], [19]. In the AFM approach, DNA is typically tethered to the probe tip through a linker-based protocol. Just like the nanopositioning system, it is difficult to limit the tethering to a single DNA strand. An alternative AFM approach that does not require tip functionalization has been investigated to achieve single DNA tethering. Here, an AFM probe is brought into contact with one end of a DNA strand deposited on a substrate to allow Van der Waals force to bind the DNA to the AFM probe. The DNA-to-probe bond strength is estimated to be about a few nanonewtons [20], [21], [22]. To enable DNA translocation, the attached DNA strand can be lifted off from the substrate by the probe so long as the DNA strand is only ‘weakly’ immobilized on the substrate. Substrate materials, such as mica and graphite, are good candidates for temporary DNA deposition. A straightforward surface treatment can create positive charges on these substrates for immobilizing negatively charged DNA molecules through a weak covalent force [23], [24], [25]. The combination of straightforward DNA immobilization and AFM manipulation provides an excellent opportunity for not only translocating a DNA strand with a high degree of control, but also delivering the DNA to another location on the substrate for detailed characterization by a sensing element.

In this report, we describe an AFM-based manipulation technique for controlling the translocation of a single DNA strand lifted off from a mica substrate and subsequently delivered to a nanoscale sensing element for electrical characterization. The DNA strand is attached to an AFM probe tip via Van der Waals force. Since the bond force between the DNA and the immobilizing substrate is lower than the DNA-to-tip force, the DNA strand can be completely lifted off the substrate. The sensing element is a 50-nm wide Pt nanoelectrode gap fabricated on a Borofloat 33 wafer. The conductance change of the nanoelectrode gap as the DNA strand translocates through the gap provides quantitative information for not only the stretching of the DNA by hydrodynamic force but also base-dependent electrical measurements across the DNA backbone that might ultimately lead to accurate sequencing of the translocated DNA.

## II. MATERIALS AND EXPERIMENTAL PROCEDURES

### A. DNA SAMPLE PREPARATION

Sample preparation began with dissolving Tris (hydroxymethyl)aminomethane hydrochloride (Tris-HCl) and ethylenediaminetetraacetic acid (EDTA) from J.T. Baker (Phillipsburg, NJ, USA) in Milli-Q water to prepare a 10 mM Tris-EDTA (TE) buffer. Potassium hydroxide (KOH) was used to adjust the pH level to 7.4. Lambda DNAs (*λ*DNAs) from New England Biolabs (Ipswich, MA, USA) were then added to the TE buffer to reach a concentration of 1 mg/mL. To enable ‘weak’ DNA immobilization on mica, 10 mM MgCl_2_ was added to the DNA sample solution prior to each immobilization event.

### B. PLATINUM (PT) NANOELECTRODE GAP FABRICATION

**FIGURE 1.**
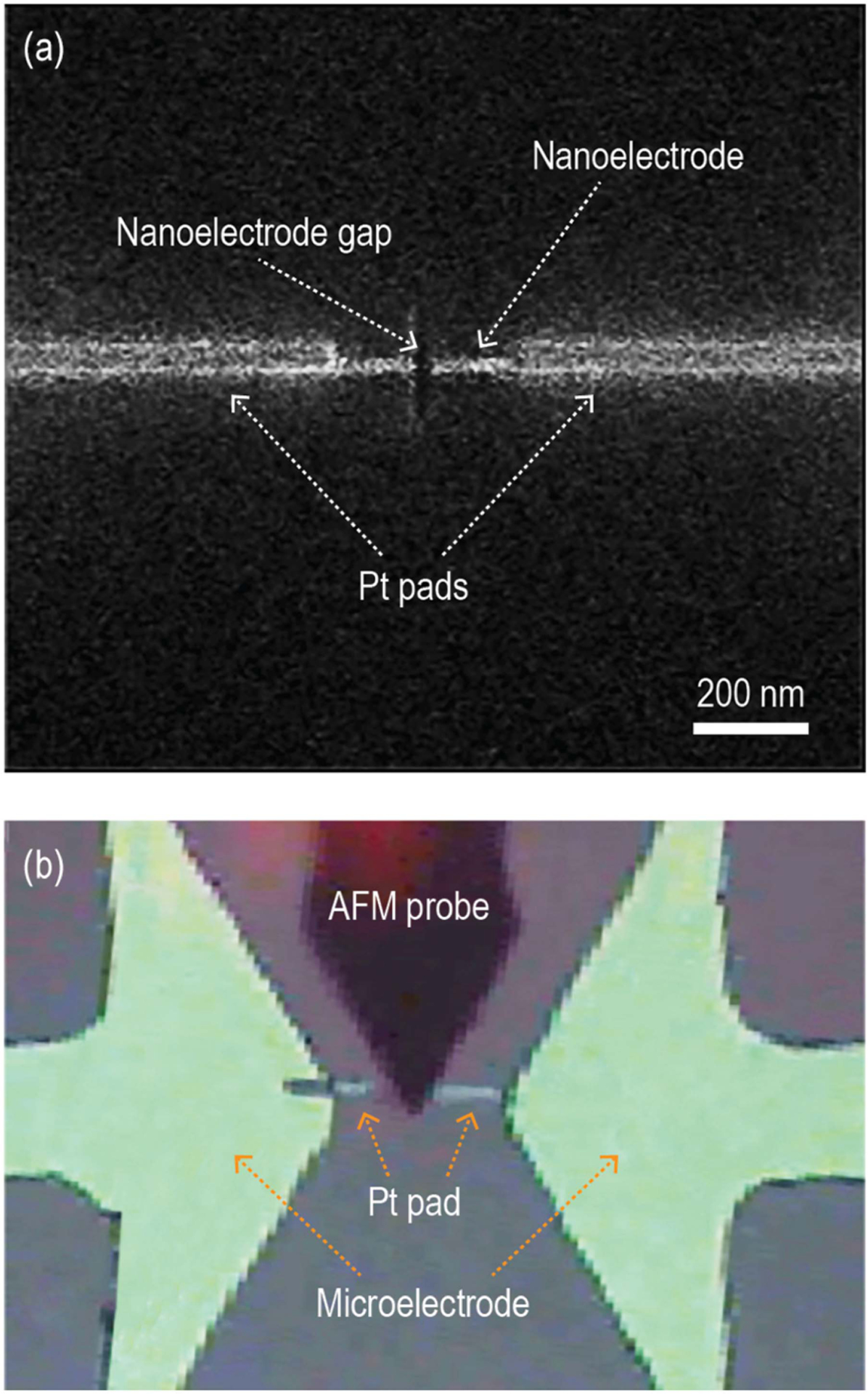
(a) Closeup image of nanoelectrode, nanoelectrode gap, and Pt pads. (b) Top view of AFM probe, Pt pad, and microelectrodes during DNA translocation process.

The fabrication process began with the deposition of Cr/Au (15 nm/45 nm) on a Borofloat 33 wafer using e-beam evaporation. Triangular-shaped microelectrodes were then patterned by photolithography. A Pt nanoelectrode gap between the microelectrodes was realized through focused ion beam (FIB) using an FEI Nova Nanolab 200 (Hillsboro, OR, USA). The FIB process consisted of two separate steps: (1) an initial deposition step where a continuous Pt nanoelectrode was deposited between the microelectrodes and (2) a subsequent milling step where a nanogap was created in the Pt nanoelectrode. The voltage and current of the FIB process were 30 KV and 30 pA, respectively. The FIB beam current was capped at 30 pA because a higher current could lead to trenching due to the competition between ion beam etching and deposition. The spatial resolution of the ion beam in the Nova system was about 15 nm, and the smallest Pt nanoelectrode achievable with high repeatability was 100 nm wide and 10 nm thick. There were two major topographical challenges in depositing the continuous nanoelectrode. Firstly, the thickness of the microelectrodes prevented the much thinner and narrower Pt nanoelectrode from achieving reliable contact with the microelectrode surface. Secondly, the 20 mm separation between the microelectrodes was too large for a continuous nanoelectrode to be realized with overcoming these challenges. Pt ‘pads’ with 500-nm thickness were first deposited to extend the reach of the microelectrodes, followed by the deposition of the 100-nm wide Pt nanoelectrode between the Pt pads. Afterward, FIB milling was used to realize the 50-nm wide nanoelectrode gap. Fig. 1 demonstrates a completed Pt nanoelectrode device and the relative position of the AFM probe during DNA translocation.

### C. DNA IMMOBILIZATION ON MICA

The process began with attaching the backside of the nanoelectrode device to a location close to the Pt nanoelectrode using double-sided tape. Next, a few layers of mica flakes were peeled off from a mica disk and attached to the double-sided tape. Immediately, 20 mL of the DNA solution was deposited onto the freshly cleaved mica surface. The immobilization process was completed by a 15-minute incubation of the deposited DNA at room temperature.

### D. EXPERIMENTAL PROCEDURE

**FIGURE 2.**
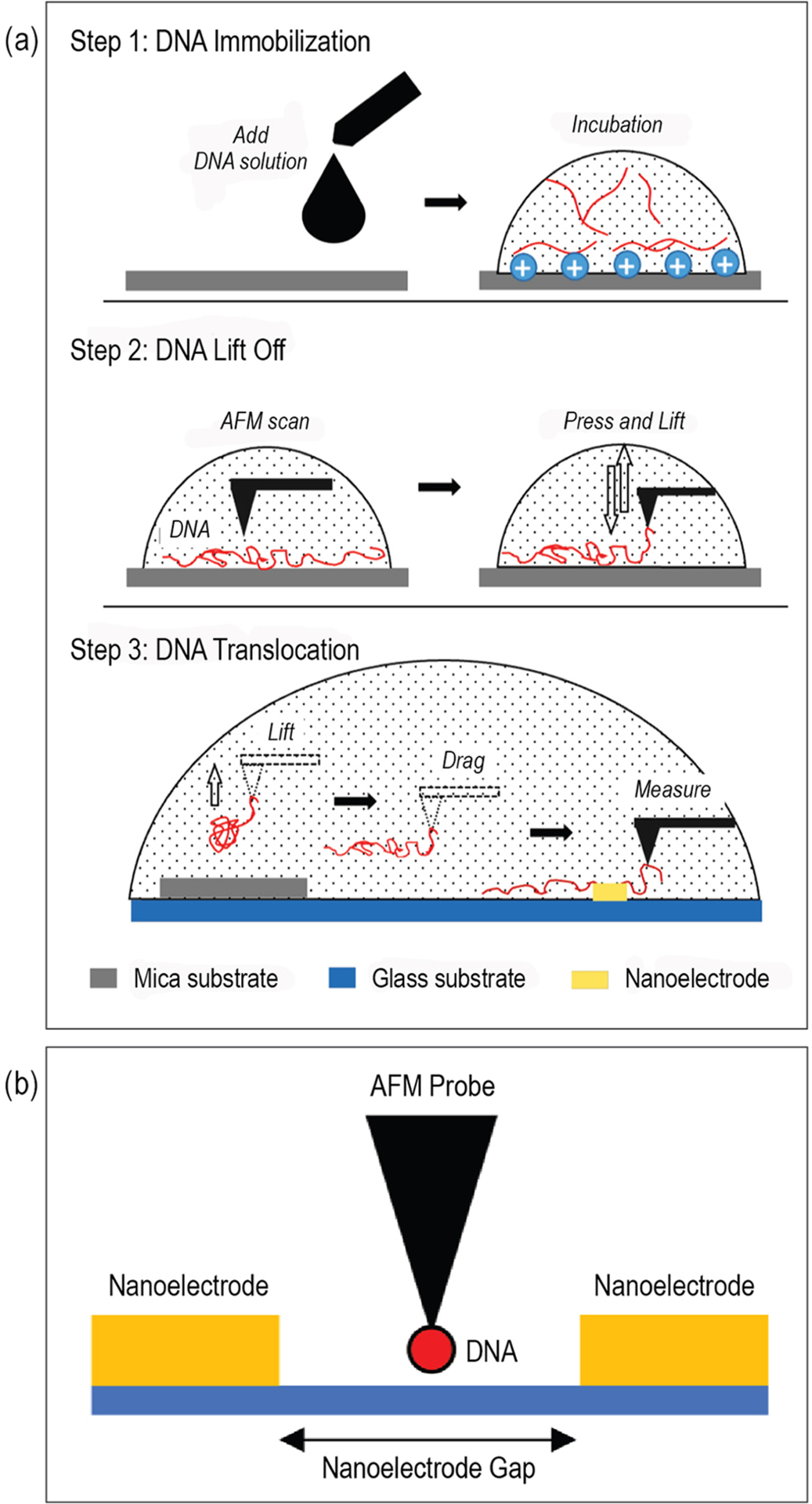
(a) Experimental procedure to translocate and electrically characterize a single DNA strand with the assistance of AFM. Step 1: DNA deposition and immobilization. Step 2: AFM working in taping mode to identify a suitable DNA strand and then in contact mode to liftoff the strand. Step 3: DNA translocation to Pt nanoelectrode gap for electrical characterization. (b) Close-up schematic of DNA translocation across the nanoelectrode gap. The translocation direction is normal to the figure. The nanoelectrodes are 10 nm thick and the nanoelectrode gap is 50 nm wide.

Fig. 2(a) demonstrates three major steps in the experimental procedure. The first step was the deposition and immobilization of DNAs on mica as described in the previous section. The second step was the use of AFM to image the immobilized DNAs and liftoff a single immobilized strand. The final step was the translocation of the freed DNA strand at a prescribed speed across the Pt nanoelectrode gap for electrical characterization as shown in Fig. 2(b). The AFM operation was conducted completely in liquid mode to avoid drying and hardening of the DNA strand.

An Agilent Scanning Probe Microscope 5500 (Santa Clara, CA) was used in the study. The AFM probe was an Arrow-CONTR probe from NanoWorld (Neuchâtel, Switzerland), which was selected due to its effectiveness in both scanning and manipulation. Tapping mode scanning of the immobilized DNA was carried out at a resonant frequency of about 7 kHz. Once a suitable DNA strand was identified, the AFM was switched to contact mode and the probe tip was re-located and suspended above the lifting point of the target DNA. To initiate the liftoff, the probe executed an ‘approach-and-retract’ process and the resultant force curve was recorded using the ‘Force Spectroscopy’ function in the AFM control software PicoView. To maintain hydration, Milli-Q DI water was added to the immobilized DNAs periodically to compensate for liquid loss through evaporation.

After the DNA strand was completely lifted off, the AFM sample stage was translocated, with the aid of an optical microscope, to move the AFM probe tip with the attached DNA strand to a small area just above the Pt nanoelectrode. Next, the probe tip was allowed to engage the substrate until the tip reached approximately 5 nm from the substrate. With a DNA strand attached to the probe tip, it was not possible for the AFM to rescan the substrate to determine the precise location of the nanoelectrode gap. To compensate for this lack of precision, the AFM was programmed to sweep the probe back-and-forth over a small area where the gap was believed to be located. Various sweeping patterns and probe speeds ranging from 1 to 20 *μ*m/s were tested to determine the optimized control parameters with which DNA translocation could take place across the nanoelectrode gap with a high degree of repeatability. To characterize the translocation process, the Pt nanoelectrodes were biased at a constant potential of 600 mV by a Keithley 2401 SourceMeter (Tektronix, Beaverton, OR, USA). A Keithley 6485 Picoammeter (Tektronix, Beaverton, OR, USA) was used to measure the resultant electrical current across the nanoelectrode gap. Digital signal from the Picoammeter was recorded by a custom designed LabView software through GPIB. All experiments were performed at room temperature.

## III. RESULTS AND DISCUSSION

### A. DNA ADHESION AND LIFTOFF

As previously described, to enable both AFM scanning and liftoff, DNA strands are ‘weakly’ immobilized on a mica surface using Mg^2+^ as the divalent cations. The immobilization force is carefully designed so that it is strong enough to resist the pushing force exerted by the AFM tip during scanning but not so strong as to prevent the DNA from being lifted off. A theoretical model is used to analyze the DNA-to-mica bonding mechanism and estimate the resultant bond strength based on a system consisting of two negatively charged planes separated by a layer of divalent cations. The negatively charged planes represent the DNA and mica substrate. The electrostatic attraction generated by the divalent cations is the primary force that immobilizes the DNA molecules. The electrostatic attractive force *F* can be determined by [26]:

(1) F(d)=e2dϵ∑i,j(1−zjφj)(1−ziφi)(xi,j2+d2)32

where *e* is the electron charge, *d* is the separation distance between DNA molecules and mica substrate, and *ϵ* is the dielectric constant of the buffer. *ϕ*_*i*_ or *ϕ*_*j*_ are the site occupation factors that can be either 1 or 0 depending on whether the site is occupied by the divalent cation or not. *x*_*i,j*_ is the distance between the *i*^*th*^ charged site on DNA and *j*^*th*^ charged site on mica. *z*_*i*_ and *z*_*j*_ represent the valence of charges of each site on DNA and mica, respectively. Based on (1), the maximum attractive force can be determined by assuming the DNA charging sites are perfectly correlated with the mica charging sites through the divalent cations. In a 10 mM Mg^2+^ buffer, the separation distance between DNA molecules and mica surface is about 0.4 nm [27]. At this distance, the attractive force calculated by (1) is 230 pN. The Van der Waals bonding force between the AFM tip and DNA is around a few nN [28], which is much higher than the predicted attractive force. As a result, the DNA should stay attached to the tip during liftoff.

**FIGURE 3.**
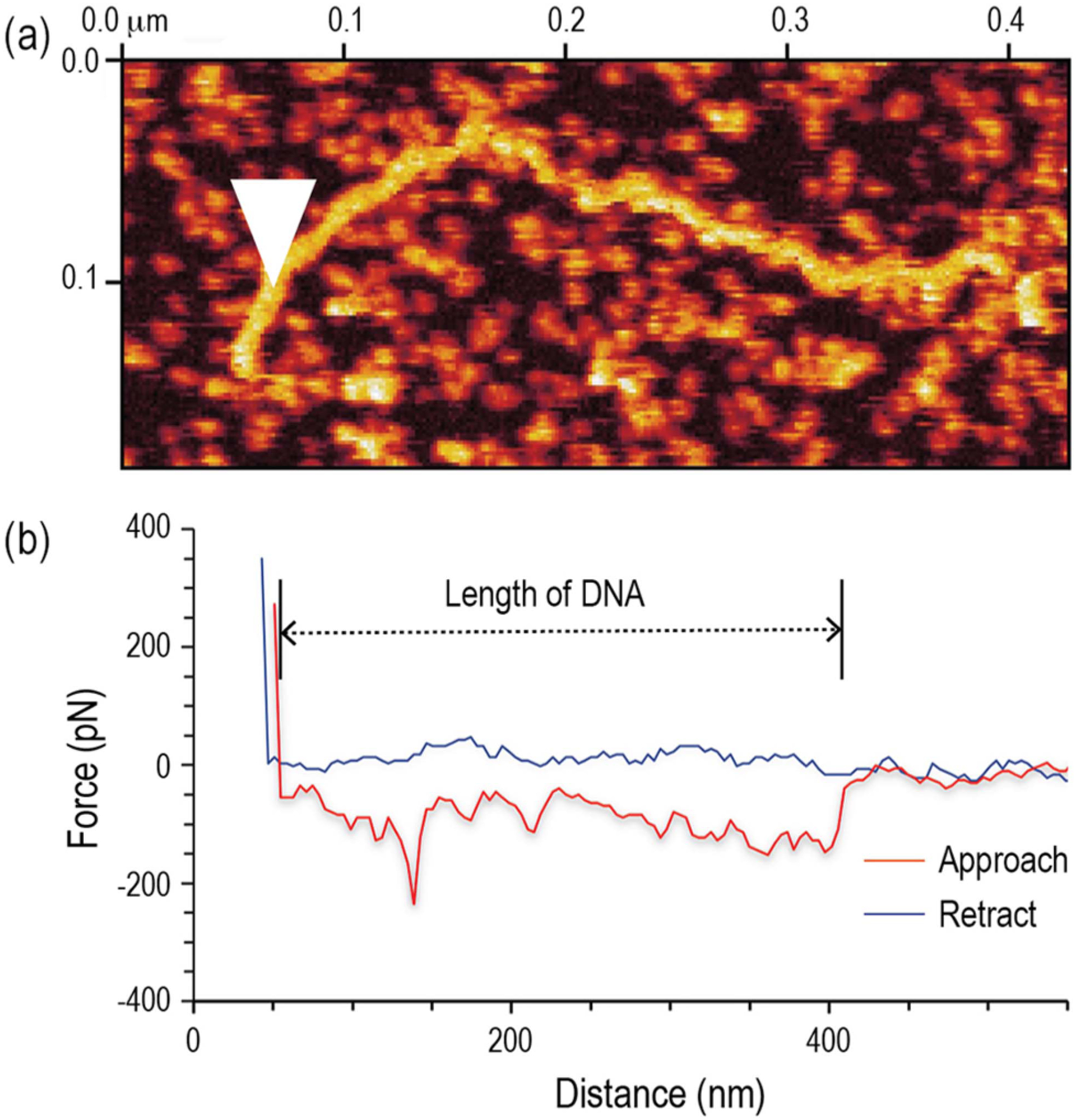
Single DNA strand liftoff using AFM microscopy. (a) AFM image of target DNA. The white triangle indicates the location where the probe tip presses down on the DNA. (b) AFM force curves recorded during the ‘approach-and-retract’ process.

The AFM probe ‘approach-and-retract’ process is successfully performed to liftoff a DNA strand from the mica substrate. As demonstrated in the pre-liftoff AFM image (Fig. 3(a)), the DNA is approximately 400 nm long and exhibits a linear, non-tangled contour, which is much more preferred than a tangled one for liftoff. The red triangle in the figure indicates the point of contact of the AFM probe. Fig. 3(b) illustrates the AFM force curves during the ‘approach-and-retract’ process. The blue line represents the close-to-zero force on the AFM probe when it travels towards the DNA on the mica surface. When it contacts the DNA, the probe is held in place for a few seconds and then retracted from the substrate. As shown by the red retractive force curve, the probe experiences an average pull-down force of about 180 pN over an upward traveling distance of about 350 nm. At the end of this distance, the pull-down disappears, and the force curve reverts to the same zero-force level experienced by the probe during the approach process. The trend of the pull-down force is a strong indication that the DNA strand has been completely lifted off from the substrate. The 180 pN required for the liftoff is somewhat lower than the 230 pN DNA-to-mica attractive force predicted by (1). A possible explanation for the discrepancy is that (1) does not consider the existence of a repulsive force that can counter the adhesion force and lower the overall liftoff force required. The repulsive force originates from a combination of electrostatic repulsion and thermal pressure. Based on the theoretical model provided in [29], a repulsive force of about 20 pN should exist between the immobilized DNA and mica substrate, leading to a revised attractive force of 210 pN. Aside from the liftoff force, a separate proof for the successful liftoff is the 350-nm probe traveling distance, which matches well with the contour length of the target DNA shown in Fig. 3(a).

### B. DNA STRETCHING AND ELECTRICAL PROPERTIES

**FIGURE 4.**
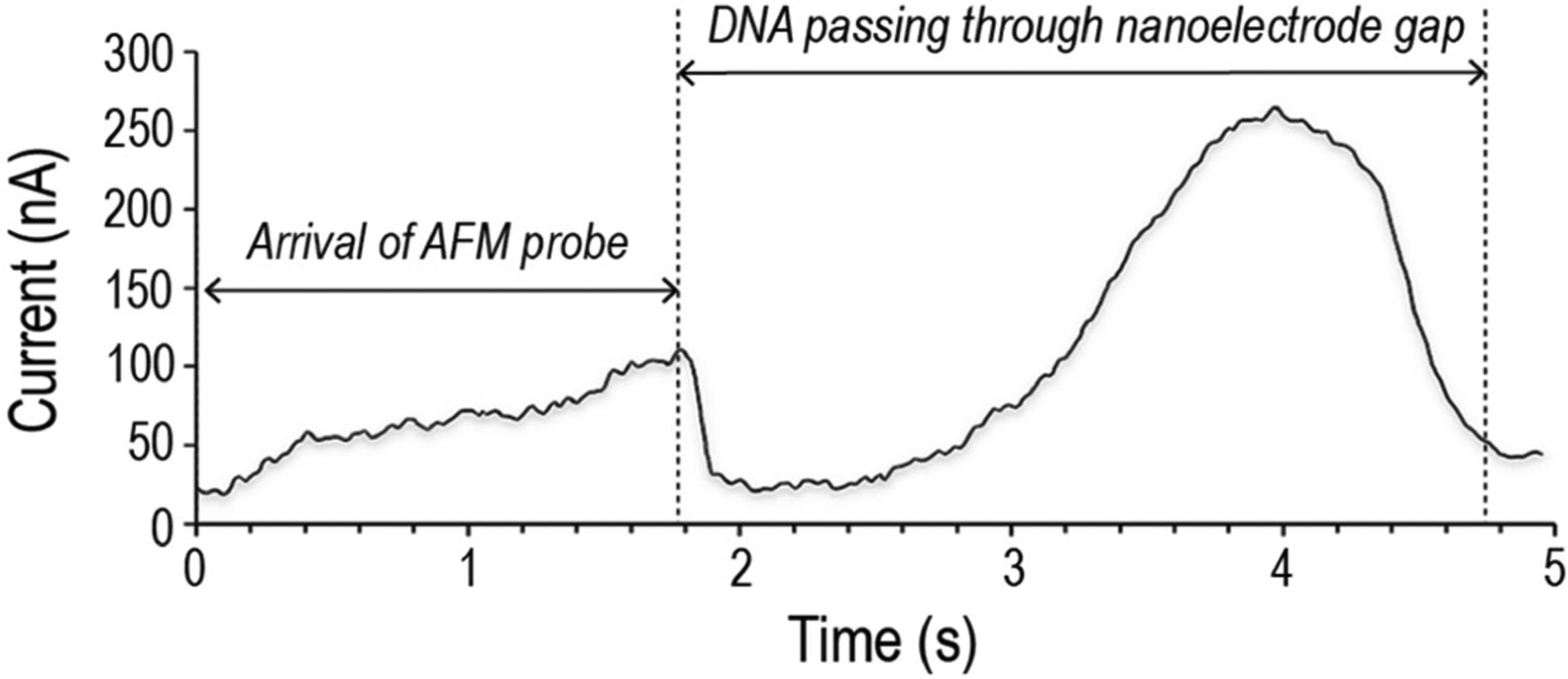
Nanoelectrode gap current during the translocation of a 16 *μ*m long DNA strand attached to a moving AFM probe.

As a DNA strand translocates through the nanoelectrode gap, the gap current is modified by the presence of the DNA bases. Fig. 4 demonstrates the continuous electrical current measured by the Pt nanoelectrodes during a typical translocation event. The DNA strand attached to the AFM probe is about 16 *μ*m long and the translocation speed (i.e., AFM probe speed) is 1 *μ*m/s. The AFM probe is maintained at a height of 5 nm above the substrate during the translocation process. This height corresponds to 1/2 of the thickness of the two nanoelectrodes that border the gap. The complete translocation event is about five seconds long and two distinct events can be identified from the measurements: (1) an initial rise in the current level indicating the arrival of the conductive AFM probe and(2) a second rise indicating the passage of the attached DNA through the gap. If the DNA strand is completely linearized during its passage, the gap current should be mostly uniform. This is obviously not the case in Fig. 4. Stretching of a tethered DNA strand in a uniform flow was previously studied using fluorescence microscopy [30]. It was shown that, while the tethered front end of the DNA was stretched by the fluid flow, the trailing free end maintained a bundled formation. The gap current in Fig. 4 demonstrates a similar behavior. The relatively consistent current level between 2–2.5s indicates the passage of a linearized DNA front end and the current ‘hump’ between 2.5–5s indicates a trailing DNA bundle. To the authors’ best knowledge, this is the first time the stretching of a tethered DNA strand is quantified by electrical measurements.

**FIGURE 5.**
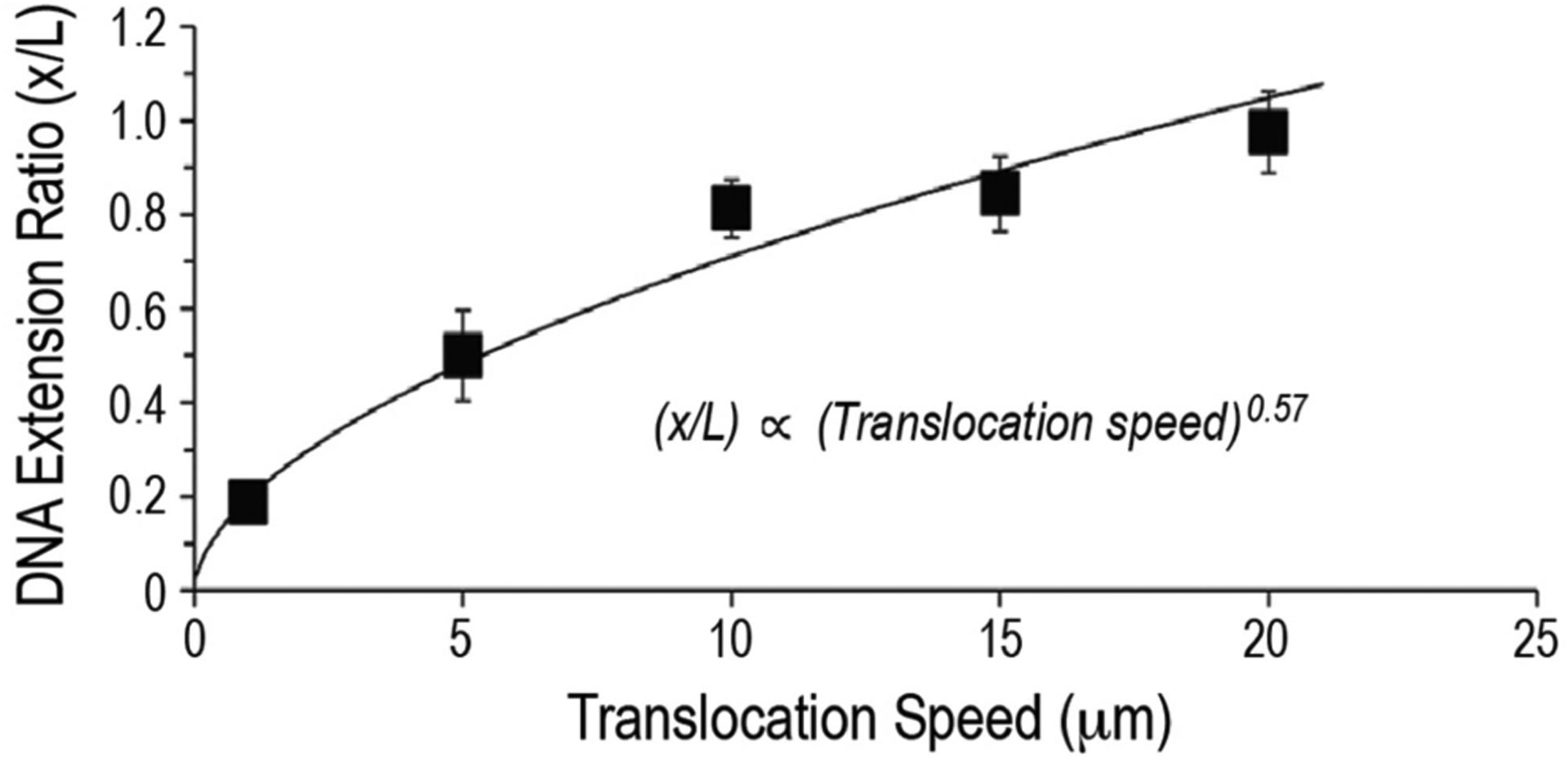
Translocation speed versus extension ratio for a DNA strand attached to a moving AFM probe.

We perform the same translocation study for a range of translocation speeds. Our results, as shown in Fig. 5, indicate the extension ratio *x/L*, where *x* is the measured DNA extension and *L* is the DNA contour length, is a function of the translocation speed *V*. Specifically, increasing the translocation speed leads to a greater degree of linearization of the DNA strand. Through curve fitting, the extension ratio *x/L* is shown to scale with *V*^0.57^. For the 16 mm long DNA used in the study, complete linearization is achieved at a speed of 20 mm/s.

**FIGURE 6.**
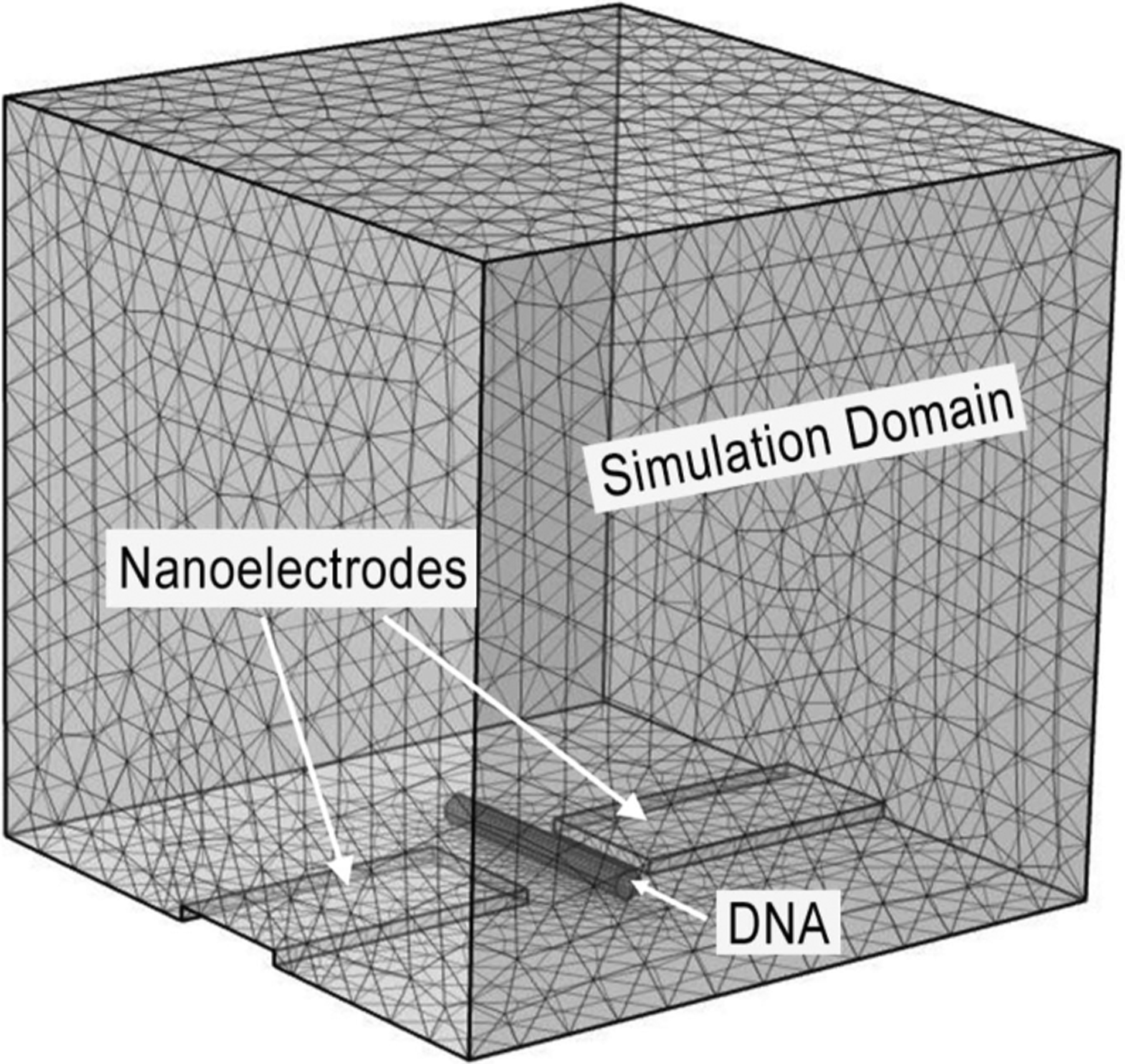
COMSOL Multiphysics simulation domain. The dimensions of the domain are 500 nm × 500 nm × 500 nm.

The nanoelectrode gap current during DNA translocation (Fig. 4) measures from 25 to 250 nA. This current level is much higher than the typical ionic current observed in nanopore-based DNA sequencing. To validate our measurements, finite element analysis using COMSOL Multiphysics (COMSOL, Inc., Burlington, MA, USA) is conducted to compute the theoretical conductivity change in the nanoelectrode gap due to the presence of a translocating DNA strand. The simulation domain of the analysis, as demonstrated in Fig. 6, consists of a nanoelectrode gap immersed in a cubic volume of MgCl_2_ buffer solution. Since the simulated electrical field is not uniformly distributed and is mostly concentrated around the nanoelectrodes, the dimensions of the cubic buffer domain are carefully selected so that the domain boundaries do not significantly affect the computed electrical property of the system. The DNA strand is modeled as a conductive cylinder with a diameter ranging from 2 to 200 nm to simulate the different DNA bundle sizes observed in the translocation study. The length of the cylinder is 180 nm. The nanoelectrodes are 100 nm wide, 200 nm long, and 10 nm thick. The nanoelectrode gap is 50 nm wide. These dimensions were chosen to match that of the FIB-fabricated nanoelectrodes shown in Fig. 1(a). The ionic mobility of Mg^2+^ is 26.53·S cm^2^/mol as suggested by [31], [32] and the relative permittivity of the buffer solution is 78 [33]. The nanoelectrodes are biased at 0.6 V.

**FIGURE 7.**
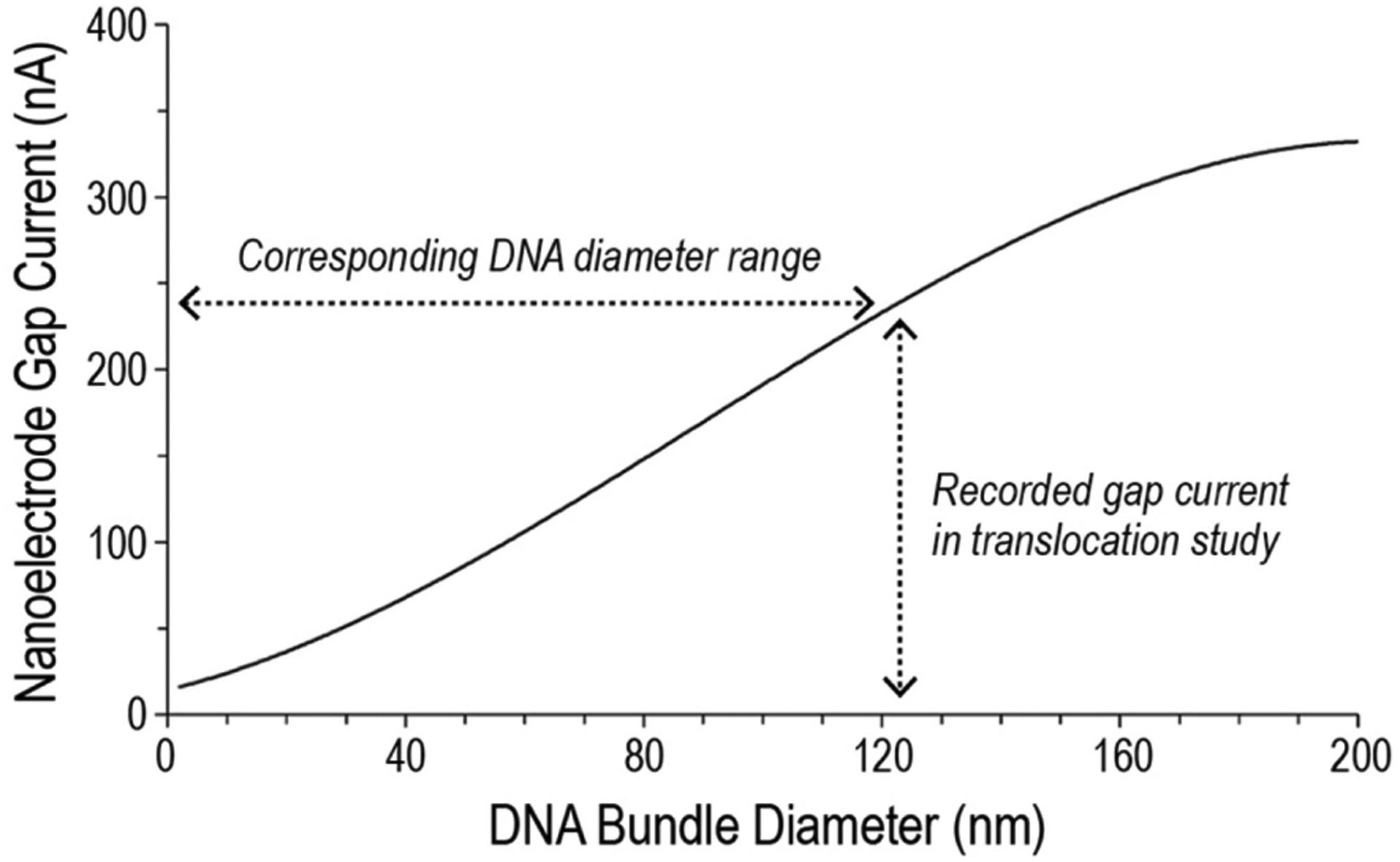
COMSOL Multiphysics simulation of DNA bundle diameter versus nanoelectrode gap current.

The COMSOL Multiphysics simulation results are shown in Fig. 7. When the simulated current is compared with the experimental gap current shown in Fig. 4, a few speculations can be made. Firstly, the experimental current range matches well with the simulated range, indicating the variation of the experimental gap current is indeed caused by the translocation of a DNA strand with a varying bundle diameter along its length. Secondly, the 25 nA gap current measured at the front end of the translocating DNA corresponds to a bundle diameter of 2 nm in the simulated result. This result is consistent with a linearized double-stranded DNA with a nominal diameter of 2 nm. Thirdly, the 250 nA gap current measured at the back end corresponds to a bundle diameter of 123 nm in the simulated result. This result also agrees well with the experimental observation. Overall, the COMSOL Multiphysics simulation provides validation for the use of a nanoelectrode gap to quantitatively characterize DNA translocations where the stretching of a tethered DNA strand is involved.

## IV. CONCLUSION

The design, fabrication, and experimental verification of an AFM-based single DNA translocation and characterization scheme is described. Through nanomanipulation, the AFM executes an ‘approach-and-retract’ process to liftoff a single DNA strand from a mica substrate where the DNA is weakly immobilized. Subsequently, the freed DNA strand is translocated by an AFM probe to pass through a Pt nanoelectrode gap where the electrical properties of the DNA are characterized. The characterization result indicates the passage of a partially stretched DNA whose degree of linearization is directly related to the translocation speed. COMSOL-based simulation is performed to validate the experimentally measured gap current. Future development of the DNA translocation and characterization scheme will focus on actual DNA sequencing, which will most likely require improvements in the precision of the ‘approach-and-retract’ process and reducing the dimensions of the sensing nanoelectrodes to better match the physical sizes of individual DNA bases.
